# Supplementary material for: Proteomic Analysis of the Predatory Venom of Conus striatus Reveals Novel and Population-Specific κA-Conotoxin SIVC
Source: Toxins (Basel). 2022 Nov 17;14(11):799. doi: 10.3390/toxins14110799 (PMC9699092; doi:10.3390/toxins14110799)
Supplement: Supplementary file 1 [file toxins-14-00799-s001.zip › toxins-2026890-supplementary.pdf]

Supplementary Materials: Proteomic Analysis of the Predatory Venom of *Conus striatus* Reveals Novel and Population-Specific κA-Conotoxin SIVC

Fabrice Saintmont, Guillaume Cazals, Claudia Bich and Sebastien Dutertre

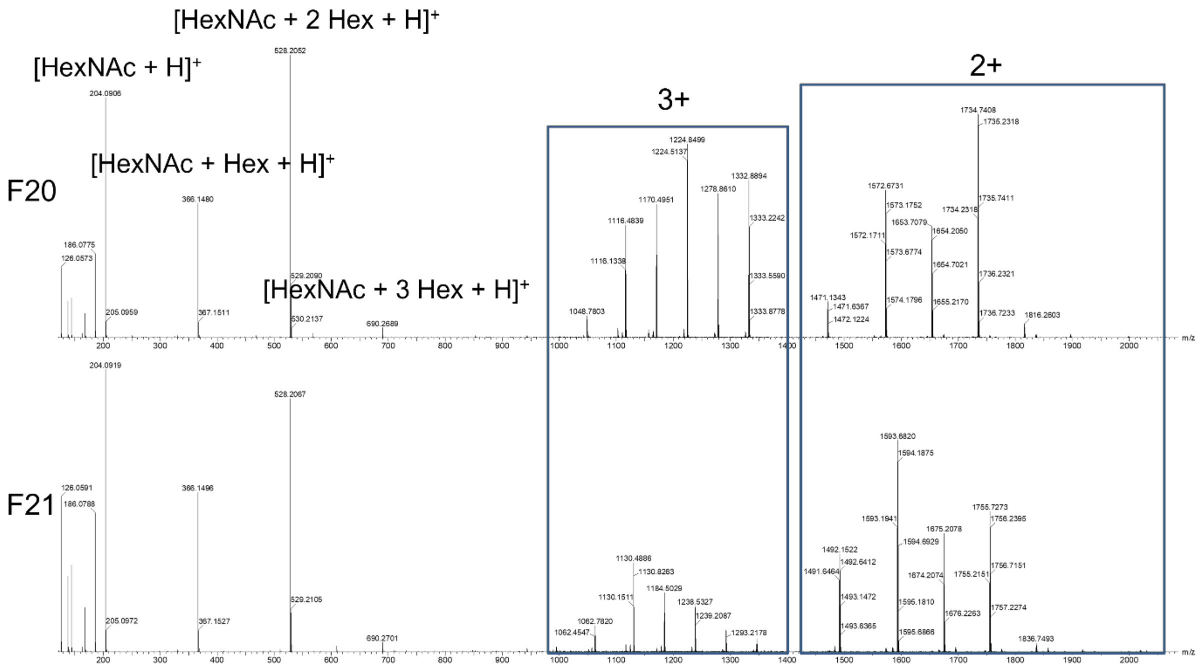

Figure S1. MSMS (CID) spectra of the 3+ peptide from the fractions 20 (m/z 1332, top) and 21 (m/z 1346, bottom) of the Conus striatus crude venom to determine the glycosylation composition.

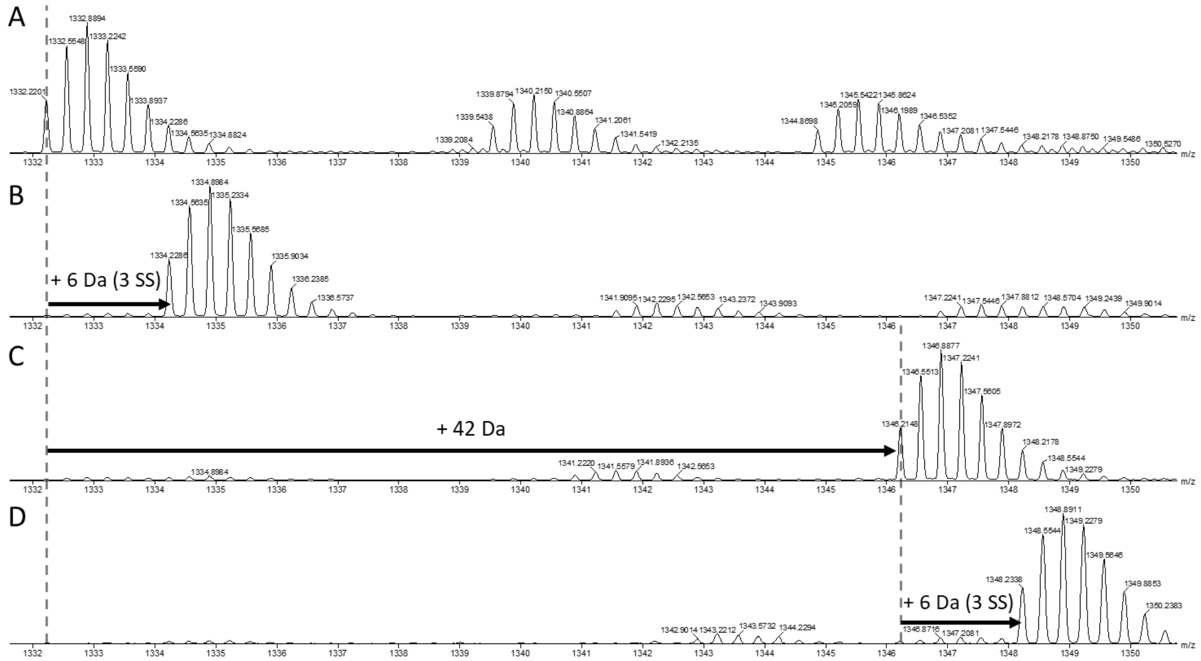

Figure S2. zoom on the 3+ ions in the MS spectra of A) fraction 20, B) reduced fraction 20, C) fraction 21 and D) reduced fraction 21.

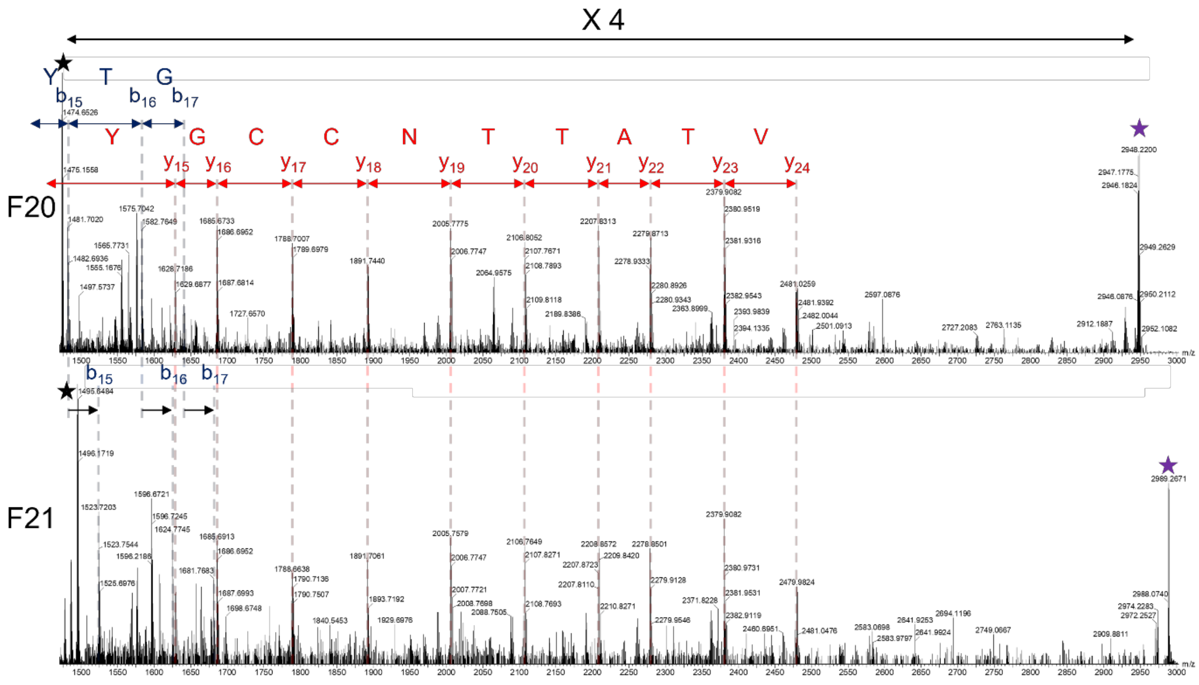

→ + 42 Da ★ Deglycosylated peptide 2+ (*m/z* 1473.64 for fraction 20 and 1494.65 for fraction 21) ★ Deglycosylated peptide 1+

Figure S3. MSMS (CID) spectra of the 3+ reduced peptides from the fractions 20 (m/z 1334, top) and 21 (m/z 1348, bottom) of the Conus striatus crude venom. The b ions are in blue and the y ions in red. Some b and y ions are not highlighted due to their low intensity.

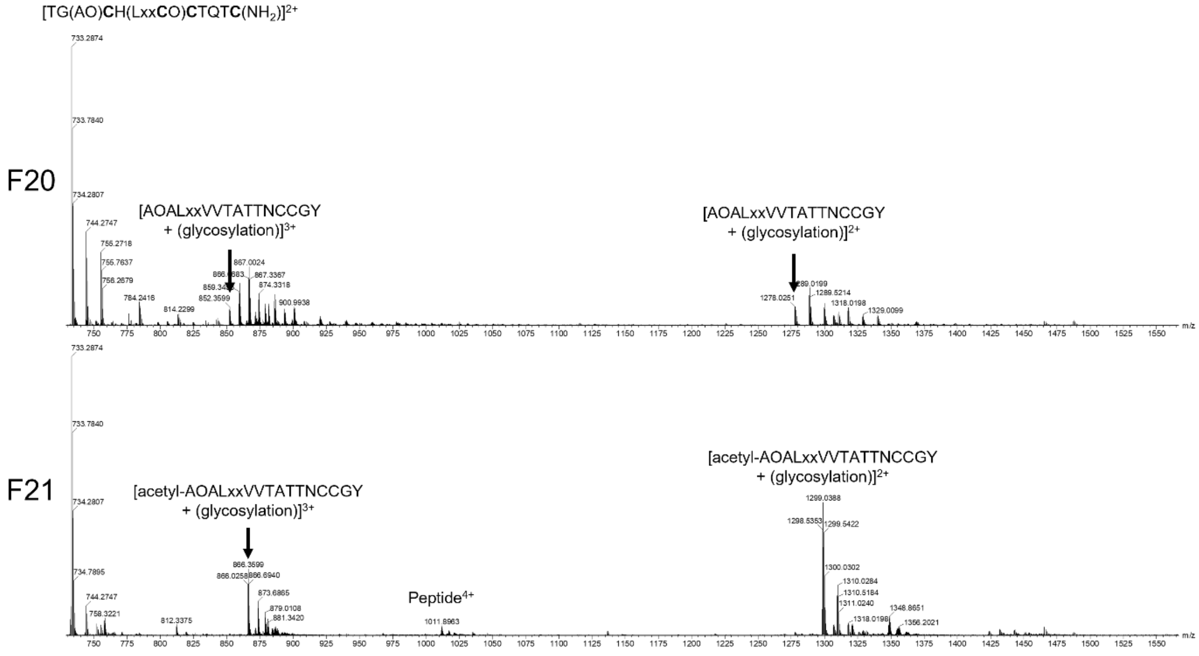

Figure S4. mass spectra of the fractions 20 (top) and 21 (bottom) of the Conus striatus crude venom digested 7 h by α-chymotrypsin.

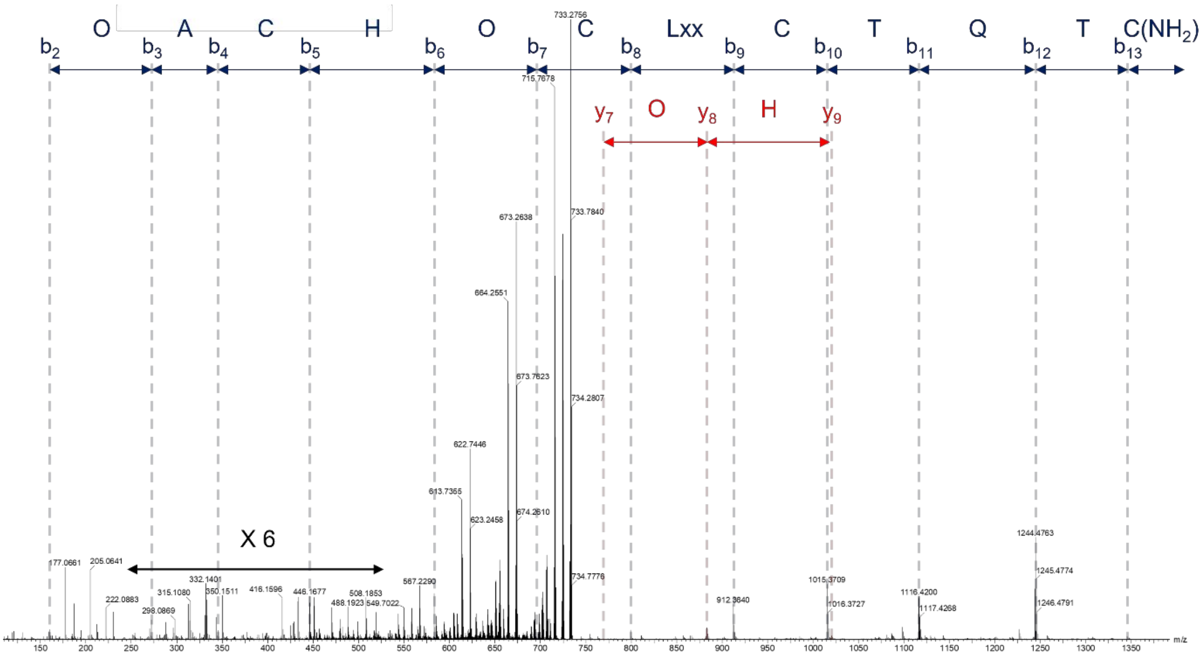

Figure S5. MSMS (CID) spectra of the 2+ reduced peptide (m/z 733.3) from the digestion by α-chymotrypsin of fractions 20/21 of Conus striatus crude venom. The b ions are in blue and the y ions in red. Some b and y ions are not highlighted due to their low intensity.

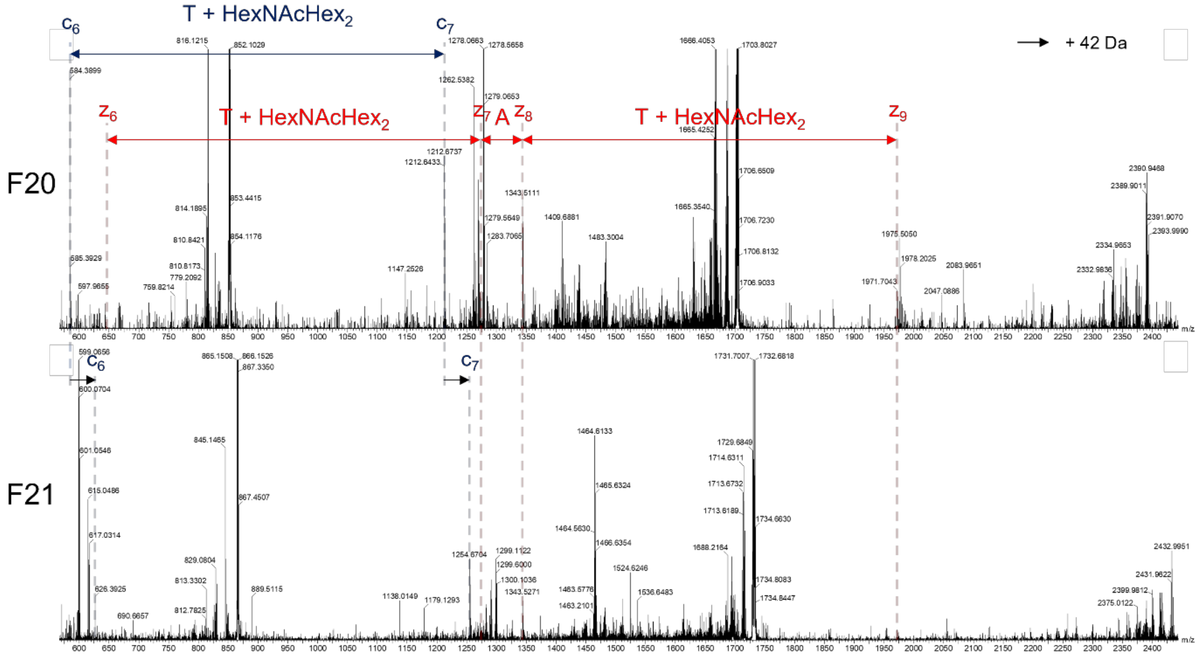

Figure S6. MSMS (ETD) spectra of the 3+ reduced peptides of fractions 20 (m/z 852, top) and 21 (m/z 866, bottom) digested by α-chymotrypsin. The c ions are in blue and the z ions in red. Some c and z ions are not highlighted due to their low intensity.

Table S1. m/z of the peptide ions and the different sugars losses for the fractions 20 and 21 from Mayotte. The shaded values are the observed ions in the MSMS spectra (Figure S1).

	F20			F21		
	**$[M+H^+]^+$**	**$[M+2H^+]^{2+}$**	**$[M+3H^+]^{3+}$**	**$[M+H^+]^+$**	**$[M+2H^+]^{2+}$**	**$[M+3H^+]^{3+}$**
Peptide	3994.6	1997.8	1332.2	4036.6	2018.8	1346.2
- Hex	3832.6	1916.8	1278.2	3874.6	1937.8	1292.2
- 2 Hex	3670.5	1835.8	1224.2	3712.5	1856.8	1238.2
- 3 Hex	3508.5	1754.7	1170.2	3550.5	1775.7	1184.2
- 4 Hex	3346.4	1673.7	1116.1	3388.4	1694.7	1130.1
- 4 Hex - HexNAc	3143.3	1572.2	1048.4	3185.3	1593.2	1062.4
- 4 Hex - 2 HexNAc	2940.2	1470.6	980.7	2982.2	1491.6	994.7
- Hex - HexNAc	3629.5	1815.2	1210.5	3671.5	1836.2	1224.5
- 2 Hex - HexNAc	3467.4	1734.2	1156.5	3509.4	1755.2	1170.5
- 3 Hex - HexNAc	3305.4	1653.2	1102.5	3347.4	1674.2	1116.5
